# Supplementary material for: Screening and Mechanism of Novel Angiotensin-I-Converting Enzyme Inhibitory Peptides in X. sorbifolia Seed Meal: A Computer-Assisted Experimental Study Method
Source: Molecules. 2022 Dec 12;27(24):8792. doi: 10.3390/molecules27248792 (PMC9785712; doi:10.3390/molecules27248792)
Supplement: Supplementary file 1 [file molecules-27-08792-s001.zip › molecules-1992743-supplementary.pdf]

# Supplementary materials

## Supplemental Table S1

**Table1 S1 Analysis of L9 $(3)^4$ test results**

| Test No. | Factors | | | | Hydrolysis degree (%) | ACE inhibition rate (%) |
|---|---|---|---|---|---|---|
| | A Hydrolysis time | B Hydrolysis temperature | C Amount of enzyme added | D Hydrolysis pH | | |
| 1 | 1 | 1 | 1 | 1 | 8.33 | 87.74 |
| 2 | 1 | 2 | 2 | 2 | 11.62 | 96.78 |
| 3 | 1 | 3 | 3 | 3 | 6.53 | 99.28 |
| 4 | 2 | 1 | 2 | 3 | 8.21 | 98.33 |
| 5 | 2 | 2 | 3 | 1 | 12.52 | 97.65 |
| 6 | 2 | 3 | 1 | 2 | 9.17 | 96.65 |
| 7 | 3 | 1 | 3 | 2 | 10.02 | 98.32 |
| 8 | 3 | 2 | 1 | 3 | 10.33 | 98.31 |
| 9 | 3 | 3 | 2 | 1 | 11.67 | 90.04 |
| Hydrolysis degree (%) | | | | | | |
| $K_1$ | 26.48 | 26.56 | 27.83 | 32.52 | | |
| $K_2$ | 29.90 | 34.47 | 31.50 | 30.81 | | |
| $K_3$ | 32.02 | 27.37 | 29.07 | 25.07 | | |
| $k_1$ | 8.83 | 8.85 | 9.28 | 10.84 | | |
| $k_2$ | 9.97 | 11.49 | 10.50 | 10.27 | | |
| $k_3$ | 10.67 | 9.12 | 9.69 | 8.36 | | |
| R | 1.14 | 2.64 | 1.22 | 2.48 | | |
| Optimal value | $A_2$ | $B_2$ | $C_3$ | $D_1$ | | |
| ACE inhibition rate (%) | | | | | | |
| $K_1$ | 283.8 | 284.39 | 282.7 | 275.43 | | |
| $K_2$ | 292.63 | 292.74 | 285.15 | 291.75 | | |
| $K_3$ | 286.67 | 285.97 | 295.25 | 295.92 | | |
| $k_1$ | 94.60 | 94.80 | 94.23 | 91.81 | | |
| $k_2$ | 97.54 | 97.58 | 95.05 | 97.25 | | |
| $k_3$ | 95.56 | 95.32 | 98.42 | 98.64 | | |
| R | 2.94 | 2.78 | 4.18 | 6.83 | | |

| | | | | |
|---|---|---|---|---|
| Optimal value | $A_1$ | $B_3$ | $C_3$ | $D_3$ |

## Supplemental Figure

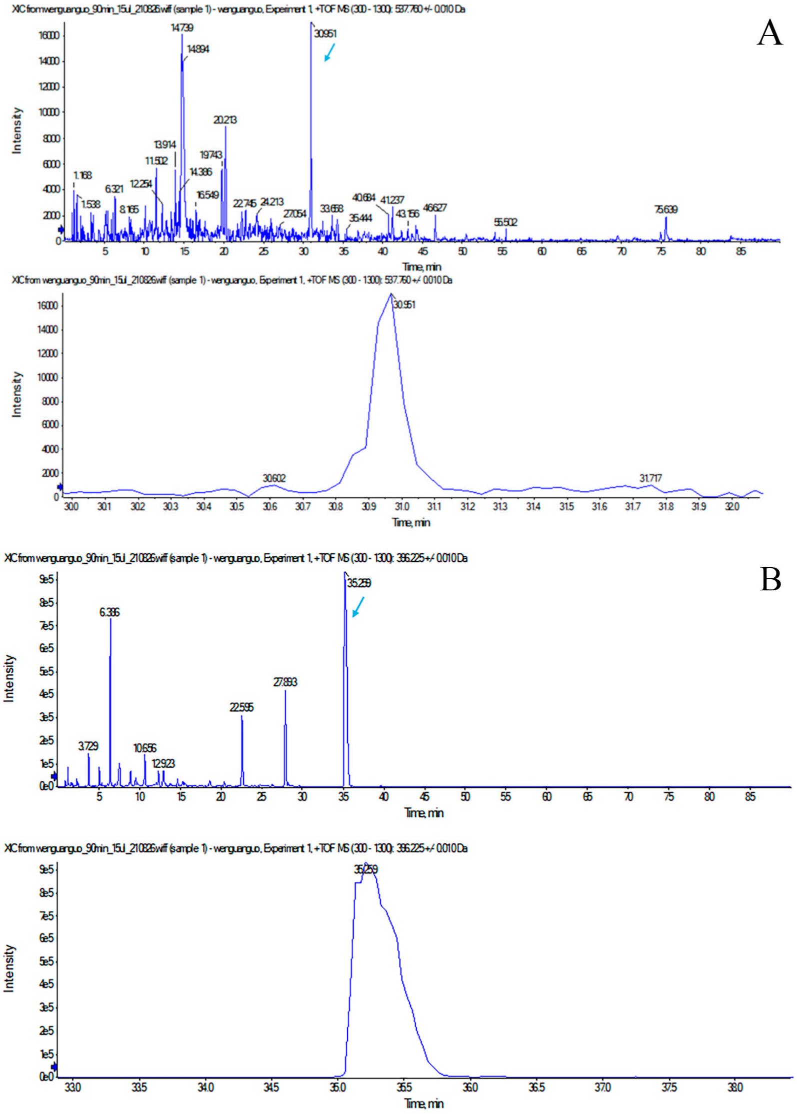

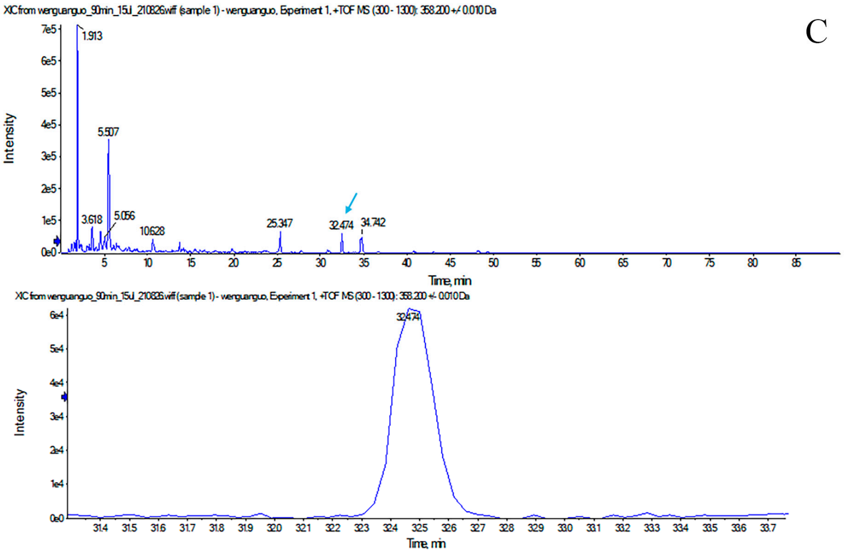
C
XIC from wenguanguo_90min_15ul_210826.wiff (sample 1) - wenguanguo, Experiment 1, +TOF MS (300 - 1300): 358.200 +/- 0.010 Da
Intensity
Time, min
1.913
5.507
3.618
5.056
10.628
25.347
32.474
34.742
32.474

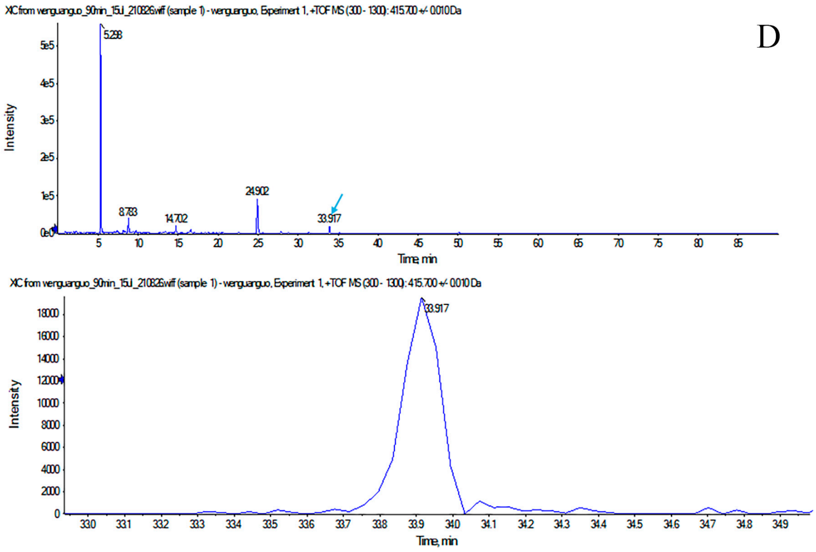
D
XIC from wenguanguo_90min_15ul_210826.wiff (sample 1) - wenguanguo, Experiment 1, +TOF MS (300 - 1300): 415.700 +/- 0.010 Da
Intensity
Time, min
5.238
8.783
14.702
24.902
33.917
33.917

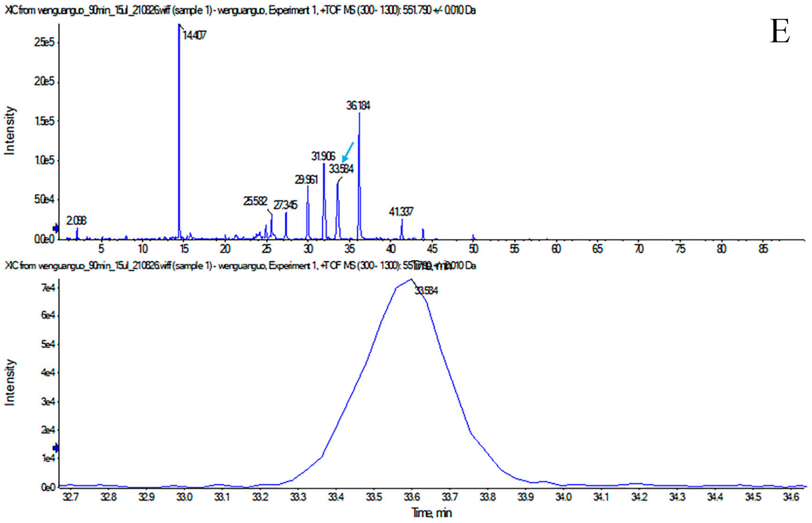
E
XIC from wenguanguo_90min_15ul_210826.wiff (sample 1) - wenguanguo, Experiment 1, +TOF MS (300 - 1300): 551.790 +/- 0.010 Da
Intensity
Time, min
14.407
36.184
31.906
33.584
29.961
25.582
27.345
41.337
2.098
33.584

F

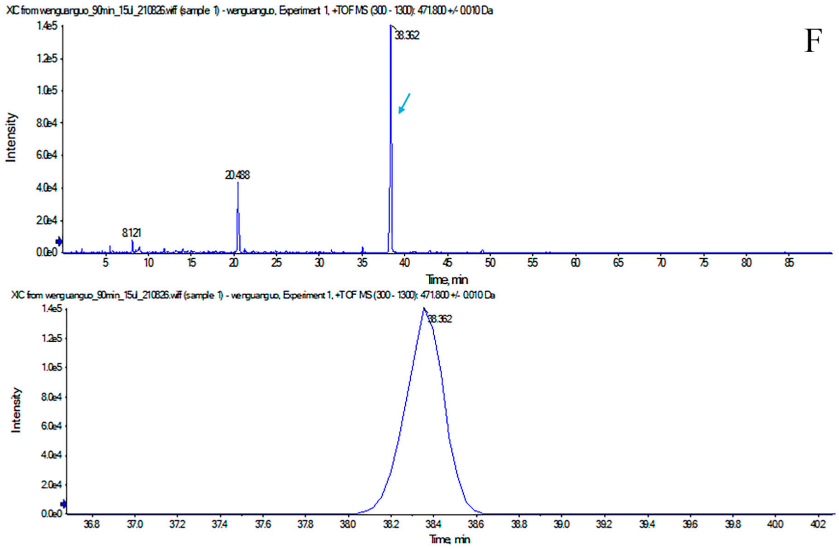
XIC from wenguanguo_90min_15ul_210826.wiff (sample 1) - wenguanguo, Experiment 1, +TOF MS (300 - 1300): 471.800 +/- 0.010 Da
Intensity
38.362
20.488
8.121
Time, min
XIC from wenguanguo_90min_15ul_210826.wiff (sample 1) - wenguanguo, Experiment 1, +TOF MS (300 - 1300): 471.800 +/- 0.010 Da
Intensity
38.362
Time, min

G

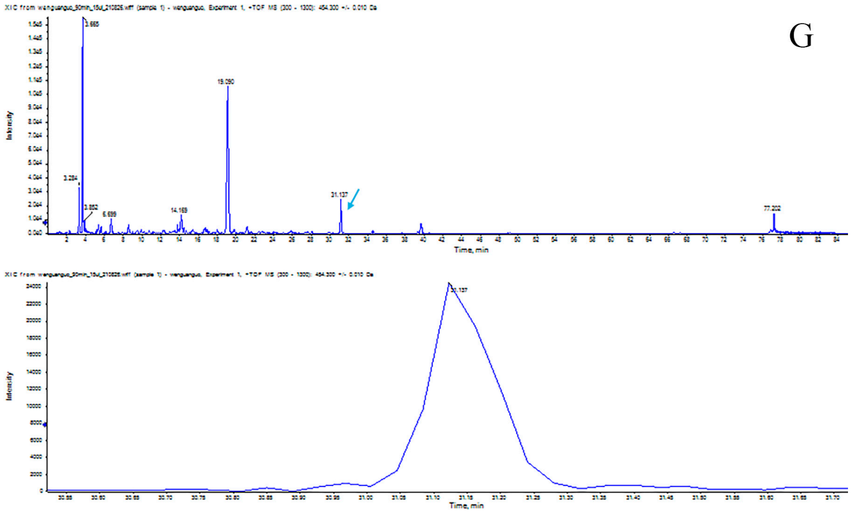
Intensity
3.665
19.090
3.284
3.852
6.699
14.169
31.137
77.202
Time, min
31.137
Time, min

H

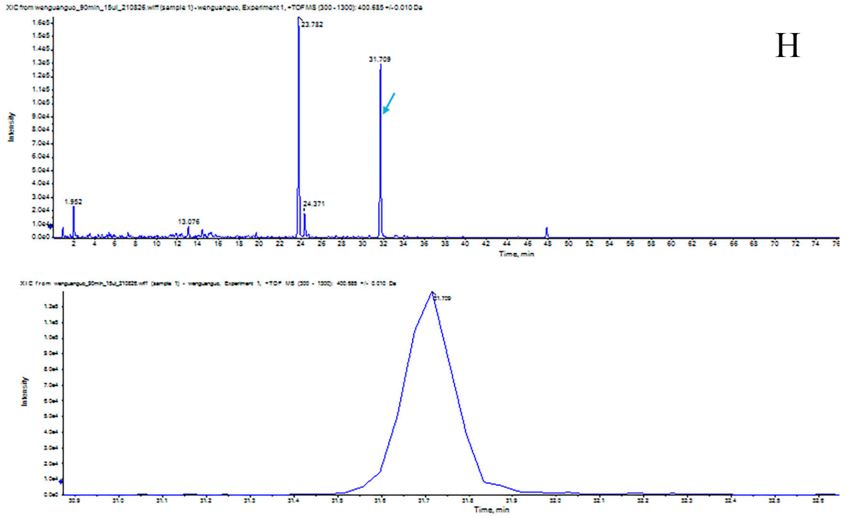
Intensity
23.782
31.709
1.952
13.076
24.371
Time, min
31.709
Time, min

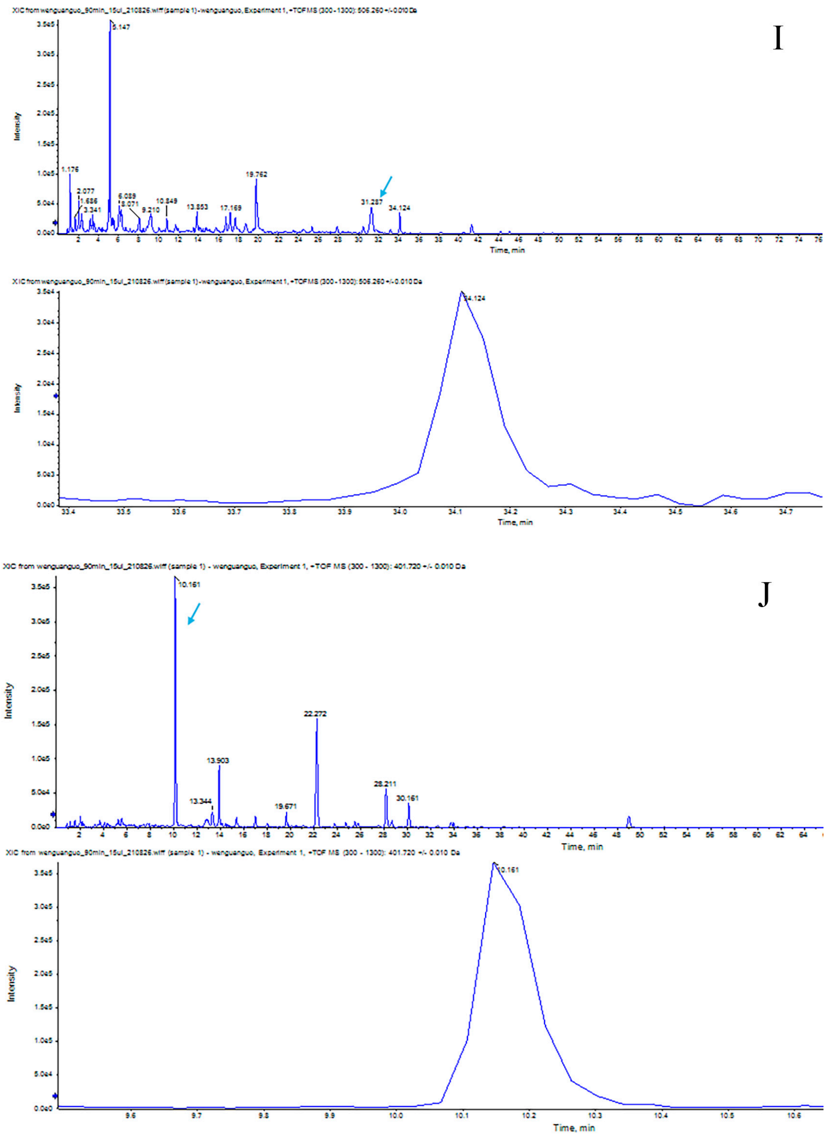


Figure S1. The ion flow diagram of polypeptide extraction from XSM. A. AEQPPLFDGT . B. GMVRELIVNVG. C. LCLELVNGVI. D. GGLPGFDPA. E. VTYPIIADPN. F. IMAVLAIVL. G. INPILLPK. H.ETYFIVR. I. TVWPGIQPN. J. IAICNGVL.
